# Identification of Loading Location and Amplitude in Conductive Composite Materials via Deep Learning Method

**DOI:** 10.3390/s26030779

**Published:** 2026-01-23

**Authors:** Zhen-Hua Tang, Di-Sen Hu, Jun-Rong Pan, Yuan-Qing Li, Shao-Yun Fu

**Affiliations:** College of Aerospace Engineering, Chongqing University, Chongqing 400044, China

**Keywords:** convolutional neural networks, loading identification, load localization, structural health monitoring

## Abstract

Current electrical self-sensing methods for composite structural health monitoring face significant limitations. Firstly, they often require complicated electrode layouts. Secondly, accurately determining both the location and amplitude of external loads remains a significant challenge. In this study, a deep learning-based self-sensing method is developed to identify the location and amplitude of external mechanical loads in resin-based conductive composites with a simple electrode layout. First, conductive filler-filled resin composites are prepared, and three-dimensional conductive networks are constructed within them. Subsequently, four electrodes are installed at the edges of the composite plate, and boundary electrical resistance responses are collected when applying mechanical loads at various positions on the composite plate. Finally, a residual learning-based CNN model is proposed for the accurate localization and amplitude identification of the applied loads. Research results demonstrate that the trained CNN model can accurately and effectively determine both the load amplitude and position. The obtained localization error and amplitude error are 0.91 mm and 0.13 N, respectively, surpassing the reported error values in previous studies. The research presented here opens a new avenue for achieving highly accurate and efficient prediction of load location and amplitude, which can be widely applied in composite structural health monitoring.

## 1. Introduction

Fiber-reinforced resin composites with the advantages of lightweight nature and excellent mechanical and thermal properties are widely utilized in aerospace, civil industries, military industries, and other fields [1,2,3,4]. In practical engineering applications, these composite materials often encounter various external loads, such as sudden impacts, which can cause invisible damage. Once the damage reaches a critical threshold, it can lead to catastrophic failures, resulting in significant economic losses and potential casualties [5,6]. Therefore, load identification and localization in service is very important to ensure the safe and effective use of composite structures [7,8]. Recently, strain gauges, fiber gratings, piezoelectric sensors, etc., have been integrated with composite materials to realize real-time healthy monitoring of composite structures [7,8,9,10]. However, these methods are often costly, complex to operate, and lack real-time monitoring capabilities [11]. Additionally, incorporating built-in sensors may compromise the material’s integrity [12]. Therefore, there is a clear need to develop a simple, efficient, and cost-effective detection method that can identify both the location and amplitude of external loads, enabling real-time assessment of the safety and performance of composite materials.

Generally, composite materials can be endowed with functional characteristics such as electrical conductivity through the addition of conductive fillers [13]. For instance, the introduction of carbon fibers (CFs), carbon nanotubes (CNTs), graphene, etc., into the composite matrix can produce self-conductive characteristics [14,15]. This allows the monitoring of stress, strain, deformation, or damage by tracking the electrical properties of the composite material [16,17,18]. For example, Alexopoulos et al. embedded CNT film into composite laminates and monitored the impact position by recording the resistance change in CNT sensing networks [16]. Since this method requires embedding a CNT sensing layer inside the laminate, the interlayer performance is weakened undoubtedly. In addition, Cheng and Tallman et al. used electrical impedance tomography (EIT) to achieve damage localization of carbon fiber-reinforced polymer (CFRP) composites [19,20]. Nonn and Rocha et al. obtained a damage localization error of 2 to 5 mm by using the EIT method with 16 electrodes [21,22]. This EIT monitoring method based on the self-electrical properties of the conductive composite material does not require additional sensors attached to the composite surface or embedded inside the composites, so it does not affect the structural strength. However, due to the inherent anisotropy and nonlinearity characteristics of composite materials, EIT monitoring technology still has problems such as ill-posedness, low resolution, large localization errors, dense electrode layouts, and substantial computational demands [23]. With the advent of the era of artificial intelligence, data-driven machine learning approaches show promise in addressing challenges such as large-scale data processing, nonlinearity, and complex pattern recognition [24,25]. For example, Kim et al. arranged multiple electrodes (five pairs) on the outer surface of CFRP composite laminates and used deep learning algorithms for damage detection and evaluation [26]. Fan et al. applied a feature fusion CNN model to realize the localization of the damage by arranging 16-channel electrodes around the CFRP composite materials [27]. Sikdar et al. developed a CNN-based monitoring method for the damage localization of CFRP using eight acoustic emission sensors [28]. Diaz-Escobar et al. combined electrical resistance tomography with several machine learning algorithms, including neural networks, random forests, k-nearest neighbors, and support vector machines, to classify and characterize damage in CFRP laminates with an accuracy of 95% [29]. Although some progress has been made in the identification of external loads by combining machine learning algorithms, previous monitoring methods usually installed a large number of measuring electrodes and collected a huge amount of data to obtain sufficient train data, which is impractical for real-world applications [19,20,21,22,26,27,30]. Furthermore, the localization errors in current studies are usually larger than 2 mm [21,22]. Most importantly, simultaneously predicting both the load amplitude and location remains challenging due to multi-parameter indentation and recognition requiring both sufficient datasets and discriminative data for each label. Therefore, it is still a great challenge to simultaneously realize the identification of external load’s amplitude and location with small prediction error via a simple electrode layout in conductive polymeric composites [31,32].

In this study, we propose a simple and effective method that combining the self-conductive properties of conductive composites and machine learning algorithms to simultaneously predict both the amplitude and location of loads in composite structures, which aims to solve the current problem of large localization errors, lack of load amplitude identification, and complicated electrode layouts [30]. Conductive carbon fillers are firstly introduced into silicone resin matrix to fabricate conductive composite materials with electrical self-sensing properties. Subsequently, a simple electrode layout strategy (four electrodes) is proposed, and boundary electrode outputs under known loading location and amplitude are collected as datasets to train a residual learning-based artificial neural network model. By combining with deep learning and the self-conductive characteristics of composite materials, high-precision identification of external mechanical loads (position and amplitude) is realized with a simple electrode arrangement layout, which provides an effective method for structural health monitoring of composite materials.

## 2. Materials and Methods

### 2.1. Design Principle 

**Figure 1:**
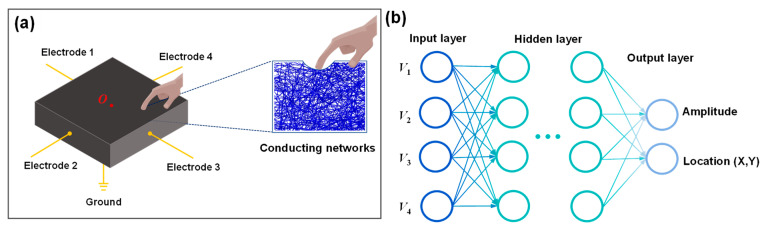
(**a**) Schematic illustration showing the electrode layout and the measurement of the resistance response characteristics of conductive composite materials. (**b**) Artificial neural network model used to predict load amplitude and location coordinates.

Figure 1a illustrates the schematic layout of the electrode configuration and the measurement of resistance response characteristics of conductive composite materials. When an external load is applied to the conductive composite, local deformation of the material occurs, resulting in changes to the position, orientation, and connectivity of the internal conductive particles (or conductive networks). This changes the conductivity of the material, exhibiting a distinct piezoresistive behavior. The mechanical loads are applied at different positions; the resistance changes in boundary electrodes will be different due to the different loading position producing a different connection status of internal conducting networks [33]. Additionally, variations in load amplitude also result in distinct resistance changes across the measurement channels. Obviously, these multi-channel response datasets are high-dimensional and contain discriminative information that preserves the key features related to both the load position and amplitude. Machine learning models, particularly deep learning algorithms, are capable of automatic feature extraction and robust processing of temporal data, making them well-suited to capture the essential characteristics of the collected datasets. Among deep learning algorithms, CNN is widely used for image recognition and analysis, specifically designed to process pixel-based image data. It can automatically capture the important features and is computationally efficient because of the convolutional and pooling layers in CNN. As shown in Figure 1b, an artificial neural network model typically consists of multiple layers, such as an input layer, one or more hidden layers, and an output layer. In this study, four-channel electrical resistance response data were collected while applying mechanical loads at various positions on the composite plate, and then these data were encoded into 2D images for training and testing the CNN model. The outputs of the model are the amplitude and location (X, Y) parameters of the applied load.

### 2.2. Materials and Characterizations

First is silicone resin (Ecoflex00-20, Smooth-On, Macungie, PA, USA); a component (4 g) was uniformly mixed with CNTs (0.6 g), and then silicone resin B component (4 g) was added into the obtained solution. Subsequently, the above mixture was thoroughly stirred, degassed, and then injected into a mold, where it was allowed to solidify at room temperature. After curing, the resulting CNT/silicone resin composite material was cut into square specimens with a size of 30 mm × 30 mm. The thickness of the specimen was 5 mm. Insulating tape was applied to the edges of the composite material specimen, creating an effective loading area of 20 mm × 20 mm. Four silver wires (electrodes) were installed along the edges of the specimen using silver pastes to minimize contact resistance, and a grounded silver wire was also bonded to the symmetric center of the specimen. 

**Figure 2:**
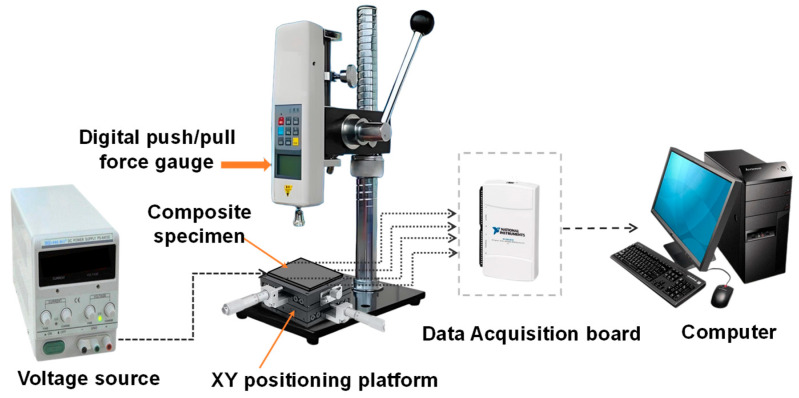
Experimental testing setup.

The measurement setup (Figure 2) mainly consists of a digital push/pull force gauge (HPA, Dongguan Fuma Electronic Equipment Co., Dongguan, China), a data acquisition system, and an XY positioning platform (LY80-C, Shenzhen Misi Precision Machinery Co., Shenzhen, China). The composite plate (specimen) was mounted on the XY stage (positioning accuracy: 0.01 mm), allowing for precise positioning at different loading locations. Quasi-static external mechanical loads, ranging from 0 to 3 N, were applied at various positions on the specimen by manually controlling the digital force gauge and adjusting the XY stage. The loading head used in the experiment was a resin cylinder with a diameter of 0.5 mm and a height of 2 cm. The corresponding resistance changes in the four electrode channels were measured and recorded using a four-channel single-arm bridge and a signal acquisition card (USB-6211, National Instruments, Austin, TX, USA). The raw signals were digitized at a sampling frequency of 1000 Hz and subsequently filtered using a third-order inverse Chebyshev filter with a cutoff frequency of 5 Hz to suppress high-frequency noise while preserving the essential load-response characteristics. LabVIEW software (2020 version) was employed for visualizing the voltage and resistance changes and for data storage.

### 2.3. CNN Model

**Figure 3:**
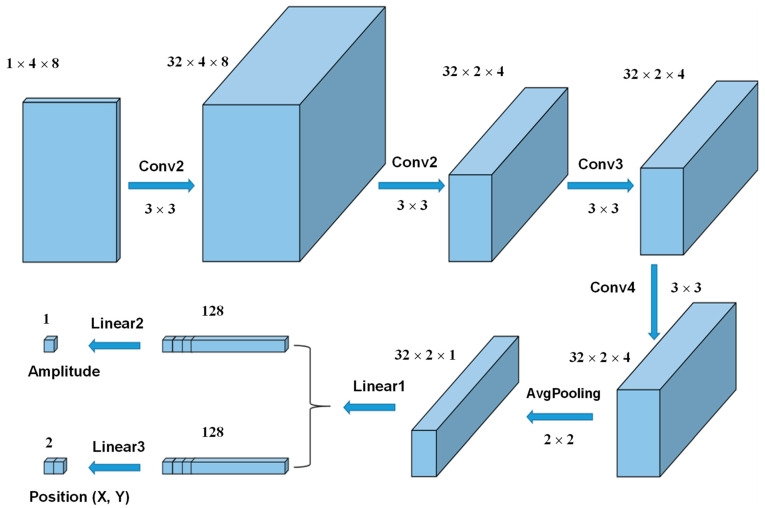
Architecture of CNN model.

CNN, as a type of artificial neural network, has strong self-learning and feature extraction capabilities [34]. This study proposed a CNN model that integrates four convolutional layers, one average pooling layer, and four fully connected layers, as shown in Figure 3. Additionally, residual learning blocks were incorporated into the CNN architecture to enhance performance. The convolution layer and pooling layer are the core components for feature extraction. The batch size of each electrode channel was set to 8, and the data of four electrode channels were spliced into a 2D image (4 × 8 matrix). The 2D image datasets were then processed using a convolution kernel (3 × 3 Sobel filter), with activation via the ReLU function at the convolution layer. The convolution kernel moves across the input matrix with a specific stride size, and the multiplied values between the input and kernel are summed to form the output. Batch normalization was applied after each convolution operation to accelerate the network training and convergence. To reduce the data size and prevent overfitting, an average pooling layer was used for downsampling. The pooling filter (2 × 2) selects the average value from each patch of the feature map, thereby reducing the spatial size of the input images. Finally, the extracted features from the 2D images were presented as planar vectors. These vectors were then fed into the fully connected layers, and their dimension was ultimately transformed at the regression layer to predict the amplitude and center coordinates (X, Y) of the loads. The output of the CNN model consists of a load amplitude matrix (8 × 1) and a loading position matrix (8 × 2). A loss function based on mean absolute error (MAE) was used to evaluate the error between predicted amplitude and true amplitude, and root mean square error (RMSE) was used to evaluate the localization error between predicted position and true position. 

**Figure 4:**
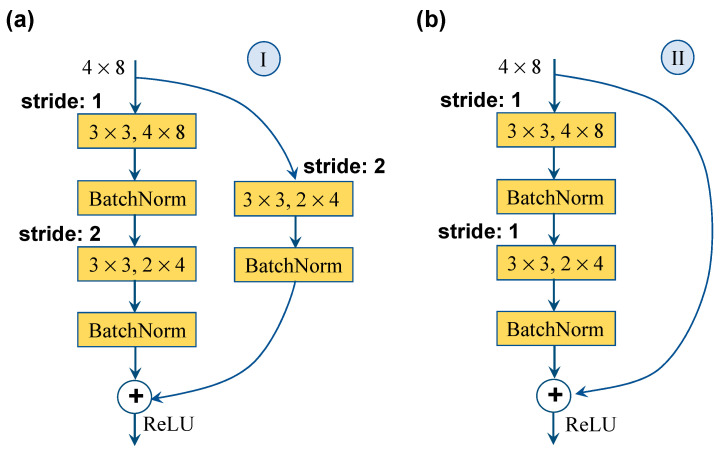
Residual blocks.

A multilayer feature analysis strategy consists of two residual blocks which are utilized to avoid the image information loss and gradient explosion. Each residual block includes two convolutional layers and one downsampling convolutional layer. To ensure consistency in the output dimension after the residual block, a judgment is performed before inputting the residual block. If the input channel differs from the output channel or the stride is not set to 1, residual block I is used, as shown in Figure 4a. Otherwise, residual block II is used, as shown in Figure 4b.

The function of the convolutional layer is used to extract the features of the 2D image datasets, and its calculation formula is as follows:

(1) $$x_{j}^{k}=f\left(\sum_{i=M_{j}}x_{j}^{k-1}\cdot w_{ij}^{k}+b_{j}\right)$$

where $x_{j}^{k}$ represents the part of the input signal to be convoluted, $M_{j}$ is the input signal, $w_{ij}^{k}$ is the convolution kernel, $b_{j}$ is the bias coefficient, and *f* is the activation function, which is used to enhance the nonlinearity of the function. The convolution kernel size of the convolution layer is 3 × 3, the padding is 1, and the stipe is 1. After the first convolutional layer, a batch normalization function is connected to accelerate training and prevent overfitting of the model. The batch normalization calculation formula is expressed as

(2) $$y=\frac{x-\mu }{\sqrt{\sigma^{2}+\varepsilon }}\gamma +\beta$$

where *μ* is the mean value of the signal under the same channel, *σ*^2^ is the variance of the signal under the same channel, *ε* is the default constant, usually 1 × 10^−5^, and *γ* and *β* are scaling and translation variables, respectively.

The activation function selects the ReLU function as follows:

(3) $$f\left(x\right)=\left\{\begin{array}{c} 0,x\leq 0\\ x,x>0 \end{array}\;\right.$$

Boundary electrical responses of each electrode were simultaneously measured during compression loading, and time series comprising one loading signal were present in one dataset.

In the DataLoader, the batch size was 8, and the shuffle was set to True. In addition, the optimizer was used to guide the parameters of the target loss function to update the appropriate size in the correct direction, so that the updated parameters make the objective function continue to approach the global minimum point. The adaptive moment estimation optimizer was selected in this study. Set the learning rate of each parameter group using a cosine annealing schedule to prevent the model from overfitting. The proposed CNN network was implemented in an NAVIDIA GPU (GTX1650, Santa Clara, CA, USA) with the open-source Python library Pytorch 2.0 and the training time was 4 h. The learning rate is set to 10^−4^.

The loss function (Smooth L1 Loss) used in this article is calculated as follows:

(4) $$loss\left(x,y\right)=\frac{1}{N}\left\{\begin{array}{c} \;\frac{1}{2}{\left(x_{i}-y_{i}\right)}^{2}\;\mathrm{if}\;\left|x_{i}-y_{i}\right|<1\\ \left|x_{i}-y_{i}\right|-\frac{1}{2}\;\mathrm{othervise}\; \end{array}\right.$$

where *N* is the number of samples, *x_i_* and *y_i_* are the real data and the prediction data, for sample *i*.

The MAE and RMSE were used to analyze the prediction results of amplitude and location of loads, respectively. MAE and RMSE are the two most common metrics for continuous variables. RMSE shows the degree of dispersion of the sample, and MAE represents the average value of the error between the predicted value and the actual value. These two parameters could be calculated as follows:

(5) $$MAE=\frac{\sum_{i=1}^{N}\left|y_{i}-x_{i}\right|}{N}$$

(6) $$RMSE=\sqrt{\frac{\sum_{i=1}^{N}{{(y}_{i}-x_{i})}^{2}}{N}}$$

## 3. Results and Discussion

**Figure 5:**
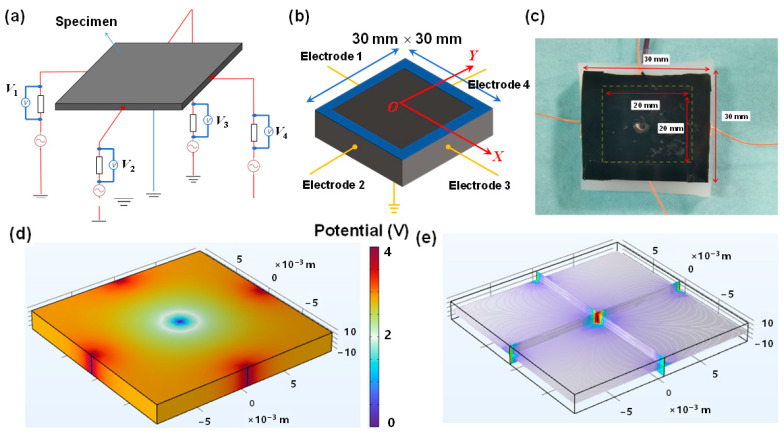
Diagram showing (**a**) the electrode configuration and (**b**) the geometric dimensions. (**c**) Optical image of the CNT/silicone resin composite. (**d**) Simulation results of the electrical potential mapping when applying a voltage of 4 V at four boundary electrodes and (**e**) the corresponding electrical field distribution.

Figure 5a,b show the schematic diagram of the electrode layout and geometric dimensions (30 mm × 30 mm) of the fabricated resin composite plate, respectively. Four electrodes were bonded to the edges of the composite plate using silver pastes, and the common ground electrode was placed at the symmetric center. Four fixed resistors (15 kΩ) were each connected in series with one of the four electrodes. A 10 V voltage was applied, and the output voltage across each fixed resistor was measured when loading. The loading region with a dimension of 20 mm × 20 mm was divided into four quadrants, and mechanical loads with various amplitudes were randomly loaded in the four quadrants (Figure 5b). Figure 5c exhibits the optical image of the fabricated CNT/silicone resin composite plate with four electrode wires. Figure 5d shows the electrical potential map obtained from the COMSOL Multiphysics (6.3 version) simulation when a voltage of 4 V was applied at the four edges of the composite specimen. The conductivity of the composite plate is set to 1.5 S/m. It can be seen that the voltage near the electrode terminals shows a higher value than that at the grounded center. Figure 5e shows the distribution of the corresponding electrical field lines. Obviously, the electrical field lines covered the entire area due to the continuous conducting network path in the fabricated CNT/silicone resin composite plate. Therefore, when external mechanical loads (such as impact force) with different amplitudes were applied at arbitrary positions of the composite plate, the electrical potential and electrical field distributions changed accordingly. This causes distinguishable resistance responses in boundary electrodes, thereby ensuring sufficient distinguishability of each label data point.

In this study, a total of 2519 samples were collected. To train the proposed CNN model, the dataset was divided into a training set (size: 2015) and a test set (size: 504) in an 80:20 ratio. In addition, it is necessary to preprocess the data before training the model for eliminating outliers and removing useless data. Specifically, 8 data points near the effective peak were selected using a Python program, which could well describe the resistance change in the composite when loads were applied. All testing was performed offline on the pre-collected and pre-partitioned test set.

**Figure 6:**
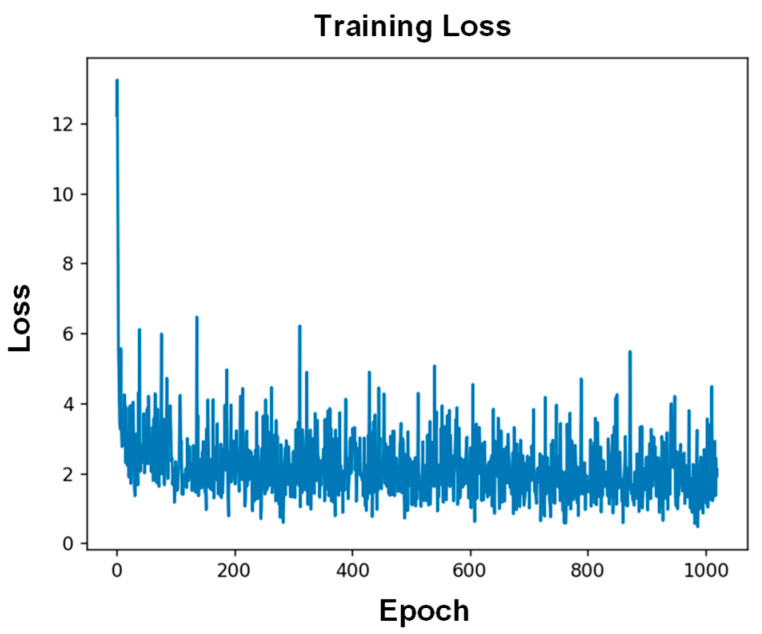
Training loss curves.

**Figure 7:**
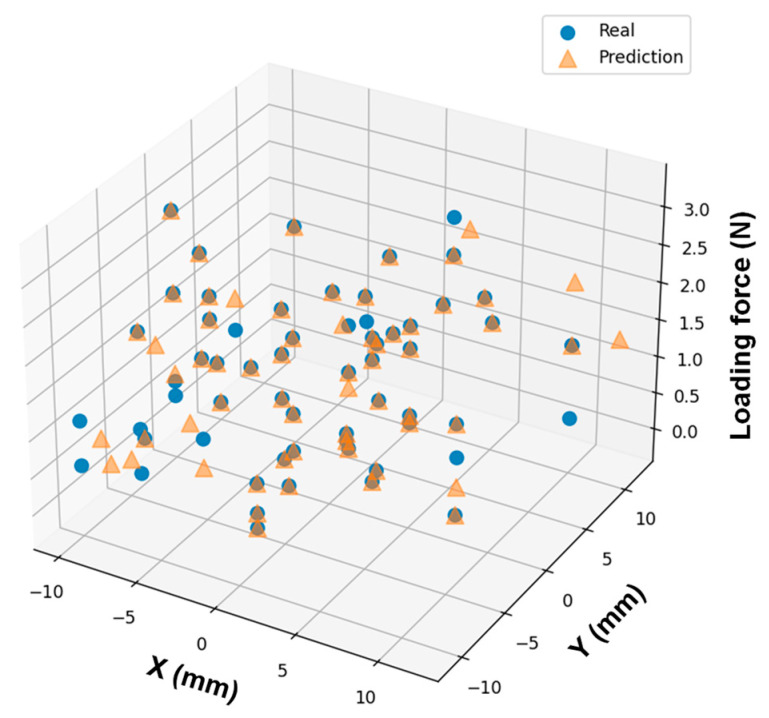
Three-dimensional scatter plot showing the prediction results and the real mechanical loading.

**Figure 8:**
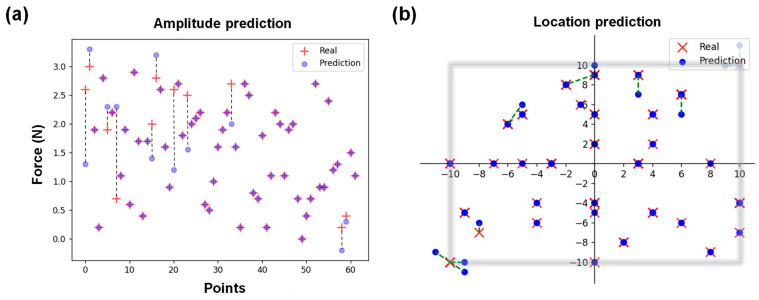
(**a**) Comparison of load amplitude predictions versus real external load, and (**b**) comparison between predicted and real loading positions.

Figure 6 shows the representative loss curves of training. It can be noted that loss of validation value converged after 10 epochs. After the first 10 epochs, the training loss curves decrease more and more slowly, suggesting convergence to a local optimal solution and developing towards lower error values. This result indicates the effectiveness of the CNN model in performing the load prediction task. A set of untrained data was input into the established CNN model to evaluate its generalization ability. All load predictions were conducted within a few seconds. Figure 7 shows the three-dimensional (3D) scatter plot of the predicted position and amplitude of the applied mechanical loads in the test set. The X-axis and Y-axis represent the position coordinates, and Z-axis is the load amplitude. It can be noted that the prediction result is consistent with experimental result for each load. To more intuitively show the predication results, two-dimensional (2D) scatter plots were used to display the real load position and their identification results. Figure 8a exhibits the prediction results for load amplitudes. The red plus sign (+) represents the real amplitude of the external loads, and the light purple round dot (●) represents the predicted amplitude. If the real value is different from the predicted value, a dotted line was used to indicate the corresponding prediction error between the two values. The calculated MAE is 0.13 N. Figure 8b shows the predicted load position coordinate (X, Y) by the proposed CNN model. The red times sign (×) represents the real load position, and the blue round dot (●) represents the predicted position. The RSME was used to evaluate the position prediction result, with the calculated RMSE being 0.91 mm. These results fully demonstrate that the proposed CNN model can learn the features of the dataset well, and exhibits an effective prediction capability with an amplitude prediction error of 0.13 N and a localization prediction error of 0.91 mm. The prediction errors in this study mainly come from the following aspects: First, electrode movement during the loading process caused fluctuations in the collected data. Second, there was some measurement error in the load position. Lastly, the sampling size of the data also has a certain effect on the prediction accuracy.

Finally, to demonstrate the advances of the proposed methods, the characteristics of this identification method were compared with those of previously reported load or damage monitoring methods, as shown in Table 1. It can be noted that the strategy proposed features the advantages in load identification and localization. This method not only exhibits smaller prediction errors for amplitude and position, but also requires significantly fewer electrodes compared to other reported methods. That means that the identification method proposed in this research can be more advanced for higher accuracy and low prediction error by using a more complicated reconstruction method. The small number of electrodes reduces the data complexity, while maintaining high accuracy in load identification and localization. It should be noted that the proposed method is currently valid only for specimens of the specific size tested (20 × 20 mm^2^). For applications to large-scale conductive composite structures like CFRPs, systematic studies will be required.

## 4. Conclusions

Current electrical self-sensing methods for damage or load identification in composite often rely on complex electrode layouts and face difficulty in achieving simultaneous localization and amplitude identification. In this study, to realize the external load localization and identification within composite structures, a new strategy based on the monitoring dataset of a limited number of electrodes around the conductive composite structure and a CNN model is proposed. Four-channel electrical responses of the fabricated CNT/silicone resin composite plate subjected to mechanical loading of varying amplitudes and positions were collected, and then the load amplitude and position were identified using a residual learning-based CNN. The model demonstrates robust generalization, with a localization RMSE of 0.91 mm and an amplitude MAE of 0.13 N. Compared to existing self-sensing nondestructive testing methods, this approach exhibits a very simple electrode layout and smaller prediction errors. Moreover, the proposed technique is particularly suited for other conductive fiber-reinforced polymer composites (such as CFRP) in applications such as wind turbine blades, aircraft, and civil infrastructure, providing an effective solution for enhancing structural safety. To realize this potential, future work should focus on real-time signal processing, electrode layout design for large surfaces, and environmental robustness.

## Figures and Tables

**Table 1:** Comparing the characteristics of this research with those of other previously reported studies.

Authors	Methods	Objectives	Number of Electrodes	Localization Errors (mm)	Amplitude Errors (Force/N)	Refs.
Lin et al.	CNN	Damage Detection	N/A	1800	N/A	[35]
Nonn and Rocha et al.	EIT	Damage localization	16	2–5	N/A	[21,22]
Yang et al.	EIT	Damage localization	16	6	N/A	[36]
Lee et al.	CNN	Damage localization	4	20–180	N/A	[37]
Jiang et al.	CNN	Damage localization	2	5.5	N/A	[30]
Tang et al.	CNN	Load localization	4	0.91	0.13	This work

## Data Availability

The data used in this manuscript are related to equipment malfunctions and are considered a significant asset of the company, making it challenging to disclose publicly. Therefore, we are unable to provide specific data supporting the results reported in this study due to privacy and ethical restrictions. We appreciate your understanding and cooperation.

## Abbreviations

The following abbreviations are used in this manuscript:

CNT	Carbon Nanotube
CNN	Convolutional Neural Network
EIT	Electrical Impedance Tomography
CFRP	Carbon Fiber-reinforced Polymer
MAE	Mean Absolute Error
RMSE	Root Mean Square Error
